# Altered TAGLN Expression During HBV Replication Correlates with OPA1 Levels

**DOI:** 10.3390/ijms27167338

**Published:** 2026-08-17

**Authors:** Juan Wen, Guoli Chen, Zhengyun Liu, Tianshun Wang, Jiarong Xie, Mingxia Xiong, Guo Luo, Huan Wang

**Affiliations:** Key Laboratory of Infectious Disease & Biosafety, Provincial Department of Education, Zunyi Medical University, No. 6 Xuefu West Road, Xinpu New District, Zunyi 563000, China; wenjuan@tmmu.edu.cn (J.W.); 18748785394@163.com (G.C.); liuzhengyun513@163.com (Z.L.); qqjwmbbpq@163.com (T.W.); 13648582018@163.com (J.X.); 18085226549@163.com (M.X.); lozens@126.com (G.L.)

**Keywords:** Hepatitis B virus (HBV), transgelin (TAGLN), mitochondria, optic atrophy 1 (OPA1)

## Abstract

Hepatitis B virus (HBV) exploits host proteins to reshape cellular physiology and metabolism, thereby supporting its replication. Transgelin (TAGLN), an actin-binding protein highly expressed in HBV-associated hepatocellular carcinoma, has a poorly defined role in HBV infection. HBV infection upregulated TAGLN expression both in vitro and in vivo. Silencing TAGLN suppressed HBV replication, increased mitochondrial fission, and elevated intracellular ATP levels. Mitochondrial proteomic analysis identified optic atrophy 1 (OPA1), a mitochondrial dynamin protein, as differentially expressed, with higher levels in HBV-positive HepG2.2.15 cells than in parental HepG2 cells. TAGLN deficiency was associated with reduced OPA1 expression. Similarly, modulation of OPA1 expression was associated with corresponding changes in TAGLN levels and HBV replication. Collectively, these findings indicate an association between TAGLN and OPA1 in the context of HBV replication. However, the precise regulatory hierarchy and molecular basis of this association remain to be determined.

## 1. Introduction

Hepatitis B virus (HBV) infection remains a major global public health challenge. Despite effective vaccination programs, approximately 296 million people worldwide live with chronic HBV infection, with serological evidence suggesting about one-third of the global population has been exposed to HBV [1]. Investigating host proteins involved in the HBV life cycle is crucial for developing novel antiviral strategies.

Cytoskeletal proteins are increasingly recognized as key facilitators of viral infections [2]. Viruses often manipulate actin reorganization to optimize their replication [3]. Transgelin (TAGLN, also known as SM22), an actin-binding protein, facilitates cytoskeletal remodeling through actin bundling [4] and functions as a cellular differentiation marker [5]. The TAGLN family includes three homologs: TAGLN2, which is widely expressed in smooth muscle cells [6], immune cells [7], etc.; TAGLN3 shows neuron-specific expression [8]; TAGLN is predominantly expressed in visceral and vascular smooth muscle [9], where it regulates myosin motor function during cellular adhesion, migration, proliferation, and inflammatory responses [10]. Aberrant expression of TAGLN2 has been reported in HBV-related hepatocellular carcinoma [11], and our preliminary data confirm TAGLN upregulation in HBV-positive samples [12], However, its precise role in HBV pathogenesis remains unexplored.

Mitochondria are highly dynamic organelles that continually undergo fission and fusion processes in response to diverse intracellular and extracellular stimuli [13]. Their interaction with the cytoskeleton not only modulates energy metabolism [14], but also maintains critical homeostasis balance [15]. While HBV infection is known to disrupt mitochondrial morphology, whether it indirectly affects mitochondrial function through host cytoskeletal proteins remains unclear. We therefore hypothesized that TAGLN, as an actin-binding protein, contributes to HBV replication by influencing mitochondrial dynamics. Using multiple HBV infection models, we demonstrate that: HBV upregulates TAGLN expression; TAGLN deficiency suppresses viral replication and alters mitochondrial morphology and energy metabolism; and mitochondrial proteomics identifies OPA1 as a differentially expressed protein associated with TAGLN-related mitochondrial changes during HBV replication. These findings provide insight into cytoskeleton–mitochondria crosstalk during HBV infection.

## 2. Results

### 2.1. HBV Promotes TAGLN Expression

**Figure 1:**
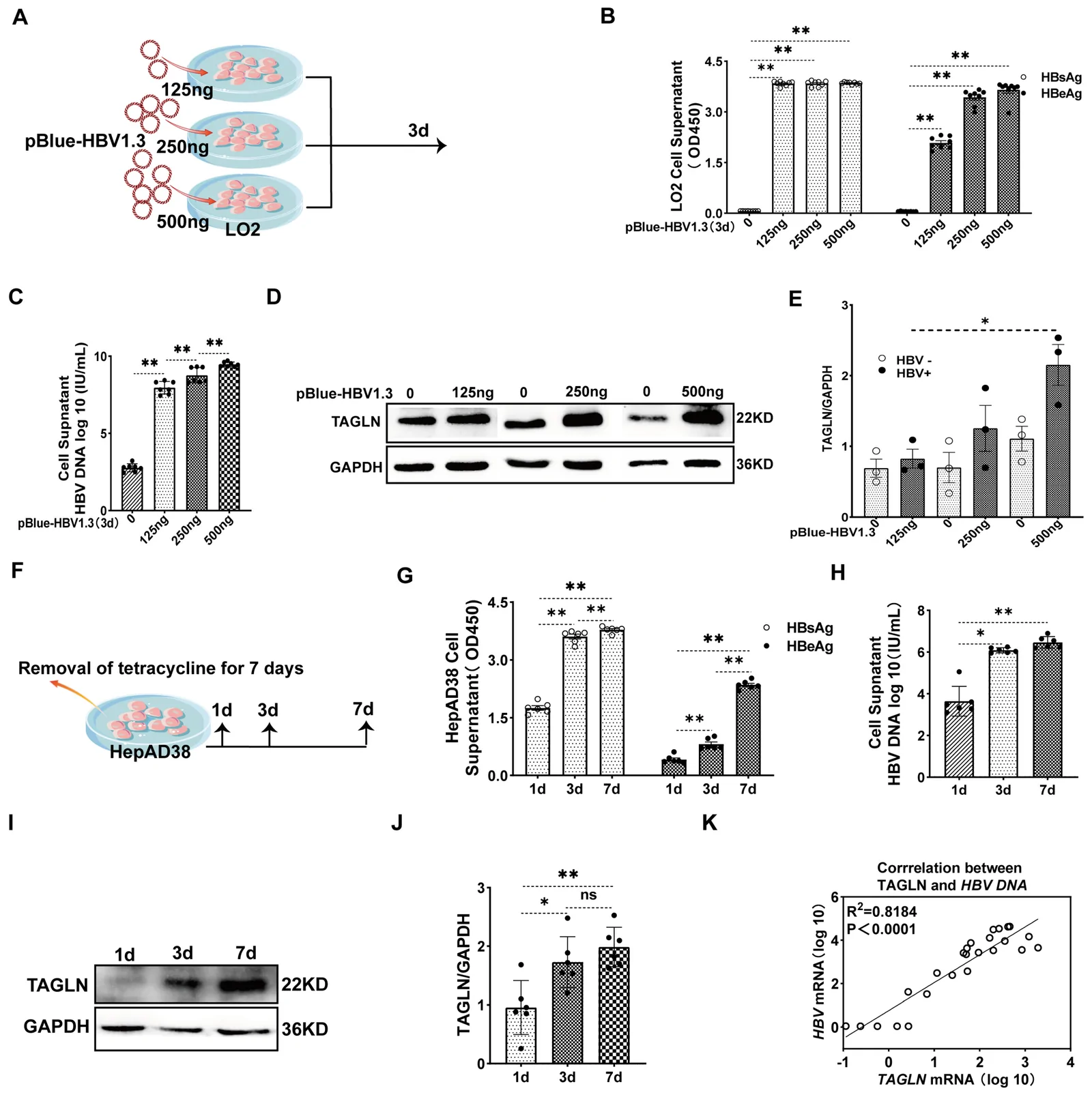
Upregulation of TAGLN expression in HBV in vitro models. (**A**) Schematic illustration of pBlue-HBV1.3 plasmid transfection in THLE-2 hepatocytes. (**B**) ELISA quantification of supernatant HBsAg and HBeAg secretion levels post pBlue-HBV1.3 transfection in THLE-2 cells. (**C**) qPCR with fluorescent probe assay measuring supernatant HBV DNA copy number following HBV transfection in THLE-2 cells. (**D**) Western blot analysis demonstrating dose-dependent TAGLN protein expression in THLE-2 cells transfected with pBlue-HBV1.3. (**E**) Statistical plot of (**D**). (**F**) Schematic of tetracycline inducible HBV replication model in HepAD38 cells. (**G**) ELISA assessment of HBsAg and HBeAg levels in supernatant after tetracycline withdrawal in HepAD38 cells. (**H**) Quantitative PCR fluorescent probe assay determining supernatant HBV DNA copy number post-tetracycline withdrawal in HepAD38 cells. (**I**) Temporal Western blot analysis of TAGLN protein expression following tetracycline withdrawal in HepAD38 cells. (**J**) Statistical plot of I. (**K**) Spearman correlation analysis between TAGLN protein levels and HBV DNA copy number, * *p* < 0.05, ** *p* < 0.01. ns, not significant.

In vitro, transfection of THLE-2 cells with increasing doses of the pBlue-HBV1.3 plasmid (Figure 1A) led to a dose-dependent upregulation of HBeAg secretion (Figure 1B) and HBV DNA replication (Figure 1C), accompanied by a progressive increase in TAGLN protein expression (Figure 1D,E). To further investigate the HBV-TAGLN relationship, we used HepAD38 cells (Figure 1F). Upon tetracycline withdrawal, HBV markers (Figure 1G,H) and TAGLN protein levels (Figure 1I,J) exhibited a concurrent rise, with a strong positive correlation between the two (Figure 1K). These findings indicate that HBV upregulates TAGLN expression, which positively correlates with viral load.

**Figure 2:**
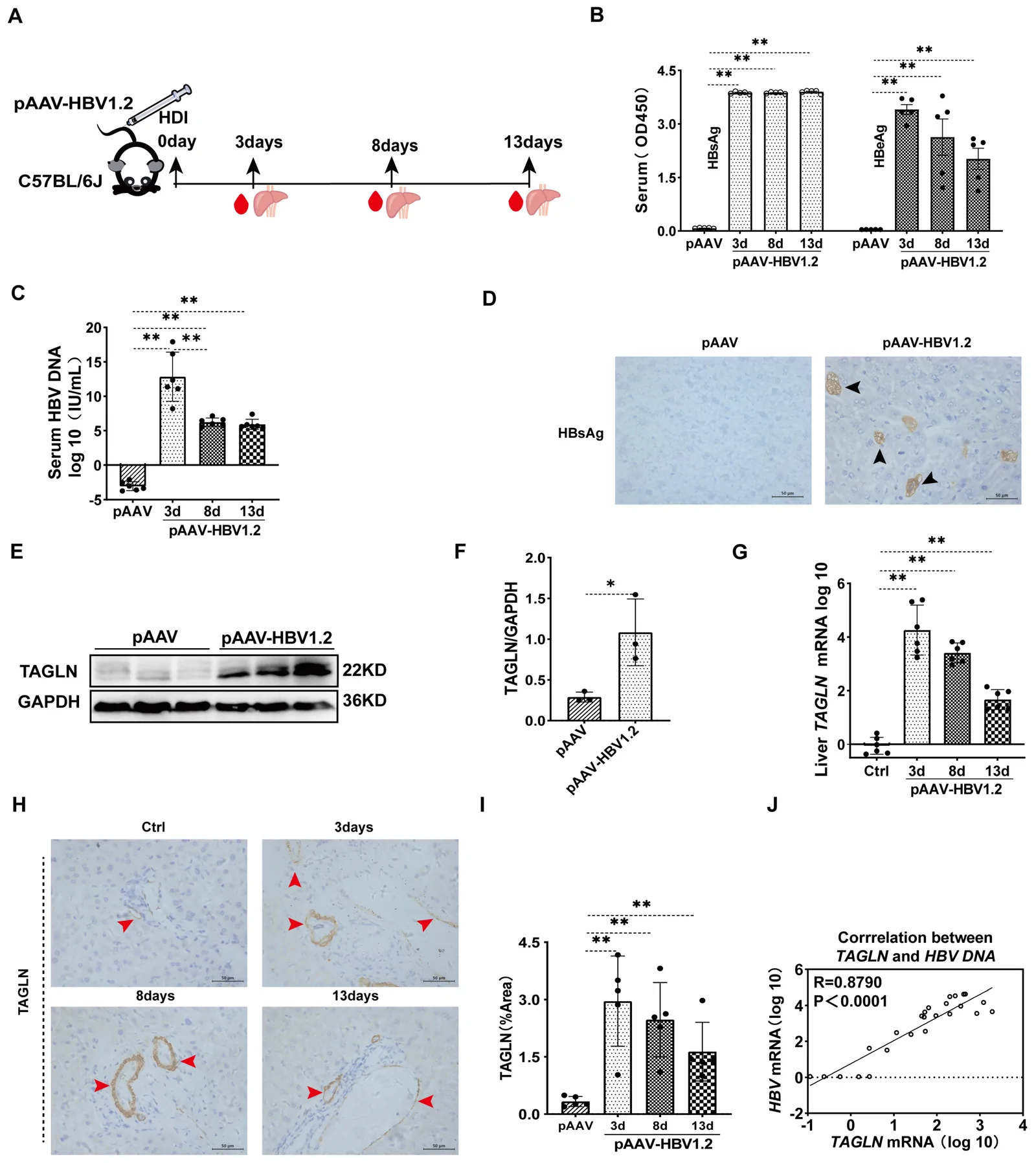
Upregulation of liver TAGLN expression in HDI mouse model. (**A**) Schematic representation of the HBV-infected C57BL/6J mouse model. (**B**) ELISA quantification of serum HBsAg and HBeAg levels; (**C**) Quantitative analysis of serum HBV DNA copies using PCR fluorescent probe assay; (**D**) IHC staining of HBsAg in liver tissues (Arrows indicate positive signals; scale bar: 50 μm). (**E**) Western blot analysis demonstrating TAGLN protein expression levels in mouse liver tissue. (**F**) Quantitative assessment of TAGLN protein expression data presented in panel (**E**). (**G**) RT-qPCR analysis evaluating TAGLN mRNA transcription levels in mouse liver tissue. (**H**) IHC staining results depicting TAGLN expression in mouse liver tissue (Arrows indicate positive signals; scale bar: 50 μm). (**I**) Quantitative analysis of TAGLN immunostaining data displayed in panel (**H**). (**J**) Spearman correlation analysis examining the relationship between TAGLN mRNA and HBV mRNA levels, * *p* < 0.05, ** *p* < 0.01.

To validate the cellular level findings, we further investigated HBV’s regulatory effect on TAGLN expression using an animal model. We established a pAAV-HBV1.2-infected C57BL/6 mouse model via HDI (Figure 2A). HBV-infected mice exhibited acute liver inflammation (Figure S1A). Successful model establishment was confirmed by HBV marker detection (Figure 2B–D and Figure S1A–D).

TAGLN protein expression was significantly elevated in the livers of pAAV-HBV1.2 injected mice compared with pAAV controls (Figure 2E,F). Analysis of liver TAGLN mRNA revealed a dynamic pattern, with peak expression observed at day 3 following injection and a gradual decline at later time points (Figure 2G). Immunohistochemical staining demonstrated that TAGLN immunoreactivity was predominantly localized to liver vascular smooth muscle cells, exhibiting strong brown yellow staining, while weaker diffuse staining was detected in liver parenchymal cells (Figure 2H,I). The temporal changes in protein expression were generally consistent with the observed mRNA dynamics. Spearman correlation analysis further showed a strong positive correlation between liver HBV levels and TAGLN mRNA expression (R = 0.8790, Figure 2J), supporting a close association between HBV replication and TAGLN upregulation in vivo.

### 2.2. TAGLN Modulates HBV Replication

**Figure 3:**
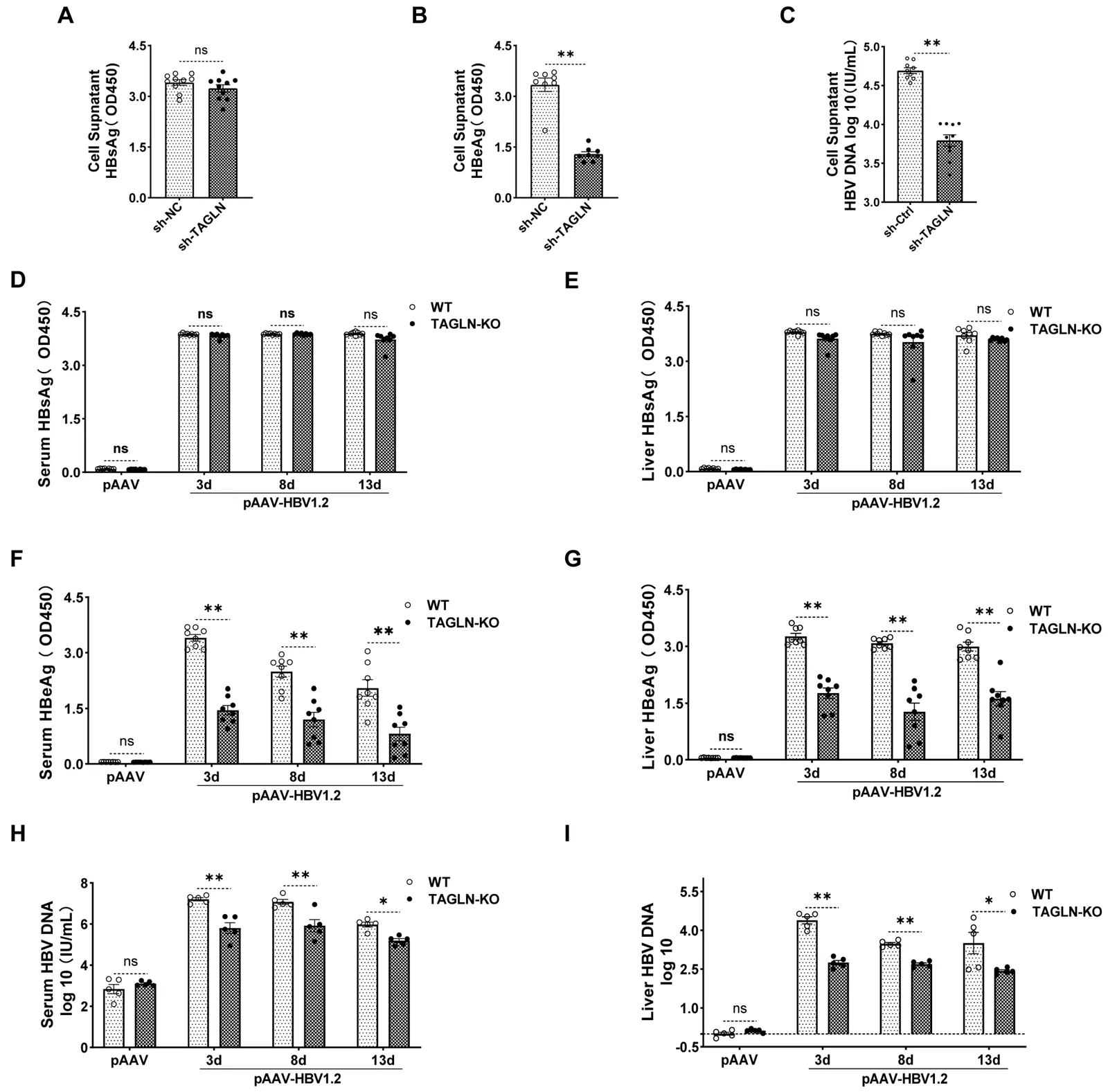
TAGLN Silencing Reduces HBV Replication. (**A**) ELISA quantification of HBsAg secretion levels in the culture supernatant of short hairpin RNA targeting TAGLN (sh-TAGLN) cells. (**B**) ELISA quantification of HBeAg secretion levels in the culture supernatant of sh-TAGLN cells. (**C**) PCR-fluorescent probe assay to quantify HBV DNA in the supernatant of sh-TAGLN cells. (**D**) ELISA detection of HBsAg secretion levels in mouse serum. (**E**) ELISA detection of HBsAg secretion levels in mouse liver tissue. (**F**) ELISA detection of HBeAg secretion levels in mouse serum. (**G**) ELISA detection of HBeAg secretion levels in mouse liver tissue. (**H**) PCR fluorescent probe assay to quantify HBV DNA copy number in mouse serum. (**I**) RT-qPCR to quantify HBV DNA copy number in mouse liver tissue, * *p* < 0.05, ** *p* < 0.01. ns, not significant.

To elucidate the role of TAGLN in HBV infection, we performed TAGLN knockdown in HepG2.2.15 cells (Figure S3). Although TAGLN silencing did not affect HBsAg secretion, it significantly decreased both HBeAg production and HBV DNA replication (Figure 3A–C). These results indicate that TAGLN deficiency suppresses HBV replication in HepG2.2.15 cells.

To further explore the role of TAGLN in vivo, we used systemic TAGLN knockout mice with a C57BL/6J background (genotyping in Figure S2A–C). Notably, TAGLN ablation did not significantly alter the expression of its isoforms (TAGLN2 and TAGLN3). Both wild-type (WT) and TAGLN-KO mice were subsequently subjected to HDI of HBV. Quantitative analysis showed no significant differences in serum and liver HBsAg levels between the two groups (Figure 3D,E), with IHC staining confirming comparable HBsAg distribution patterns in liver tissues (Figure S4). However, TAGLN-KO mice exhibited significantly lower serum HBeAg levels and reduced HBV DNA viral loads at all timepoints compared to WT controls (Figure 3F–I). Collectively, our data show that TAGLN is associated with HBV replication.

### 2.3. TAGLN Mediates Mitochondrial Structural and Functional Alterations During HBV Infection

**Figure 4:**
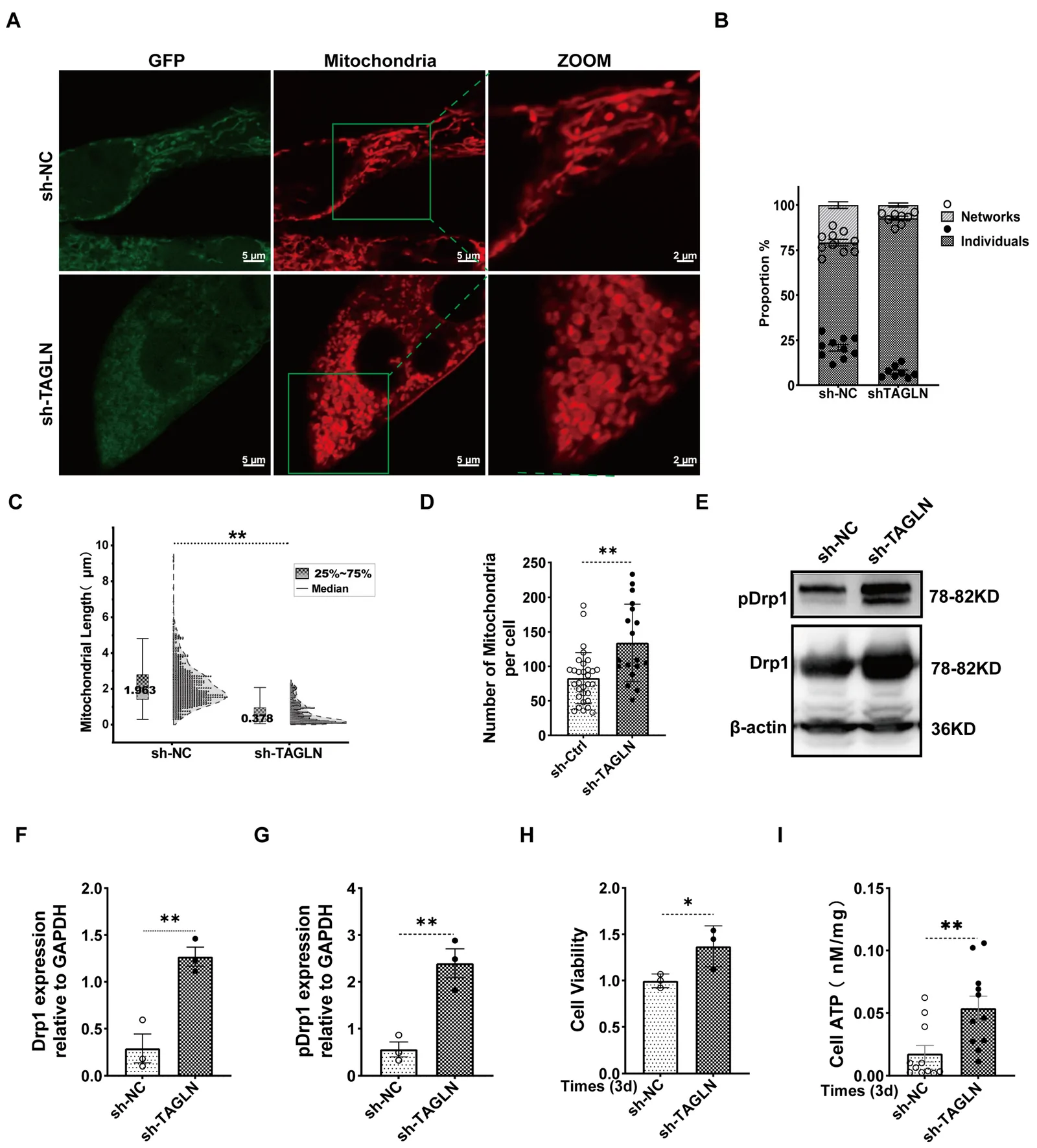
TAGLN knockdown alters mitochondrial morphology and energy metabolism in HBV-infected cells. (**A**) Confocal microscopic visualization of mitochondrial morphology in sh-TAGLN cells. Mitochondria were labeled with Mito Tracker Red (red), and GFP (green) served as a transfection marker. (**B**) Quantification of the proportion of fused (networked) versus fragmented mitochondria from the cells shown in panel (**A**). (**C**) Statistical analysis of average mitochondrial length in the cells from panel (**A**). (**D**) Quantification of mitochondrial number per cell from panel (**A**) (Scale bar: 2 μm). (**E**) Western blot analysis of Drp1 and pDrp1 protein expression in sh-TAGLN cells. (**F**) Quantification of Drp1 protein expression levels from panel (**E**). (**G**) Quantification of pDrp1 protein expression levels from panel (**E**). (**H**) Cell proliferation viability assessed by CCK-8 assay in sh-TAGLN cells. (**I**) ATP levels measured by luminometer in sh-TAGLN cells, * *p* < 0.05, ** *p* < 0.01.

This study found that HBV infection increased TAGLN expression, while TAGLN deficiency impaired HBV replication. Given that HBV infection causes abnormal mitochondrial morphology and function in host cells [16], and considering that the cytoskeleton drives mitochondrial morphological changes while TAGLN is a cytoskeleton regulatory protein, we hypothesized TAGLN may be involved in this process. Our results demonstrate that mitochondria in the control group exhibited an elongated, networked distribution. In contrast, the sh-TAGLN group showed fragmented and punctate mitochondria (Figure 4A–C), along with an increased mitochondrial number (Figure 4D) and upregulated expression of the mitochondrial fission protein Drp1 (Figure 4E–G). Functionally, sh-TAGLN cells displayed enhanced proliferation (Figure 4H) and elevated ATP production levels (Figure 4I). These findings suggest that knocking down TAGLN promotes mitochondrial fission, accelerates cell proliferation, and enhances mitochondrial function in HepG2.2.15 cells.

**Figure 5:**
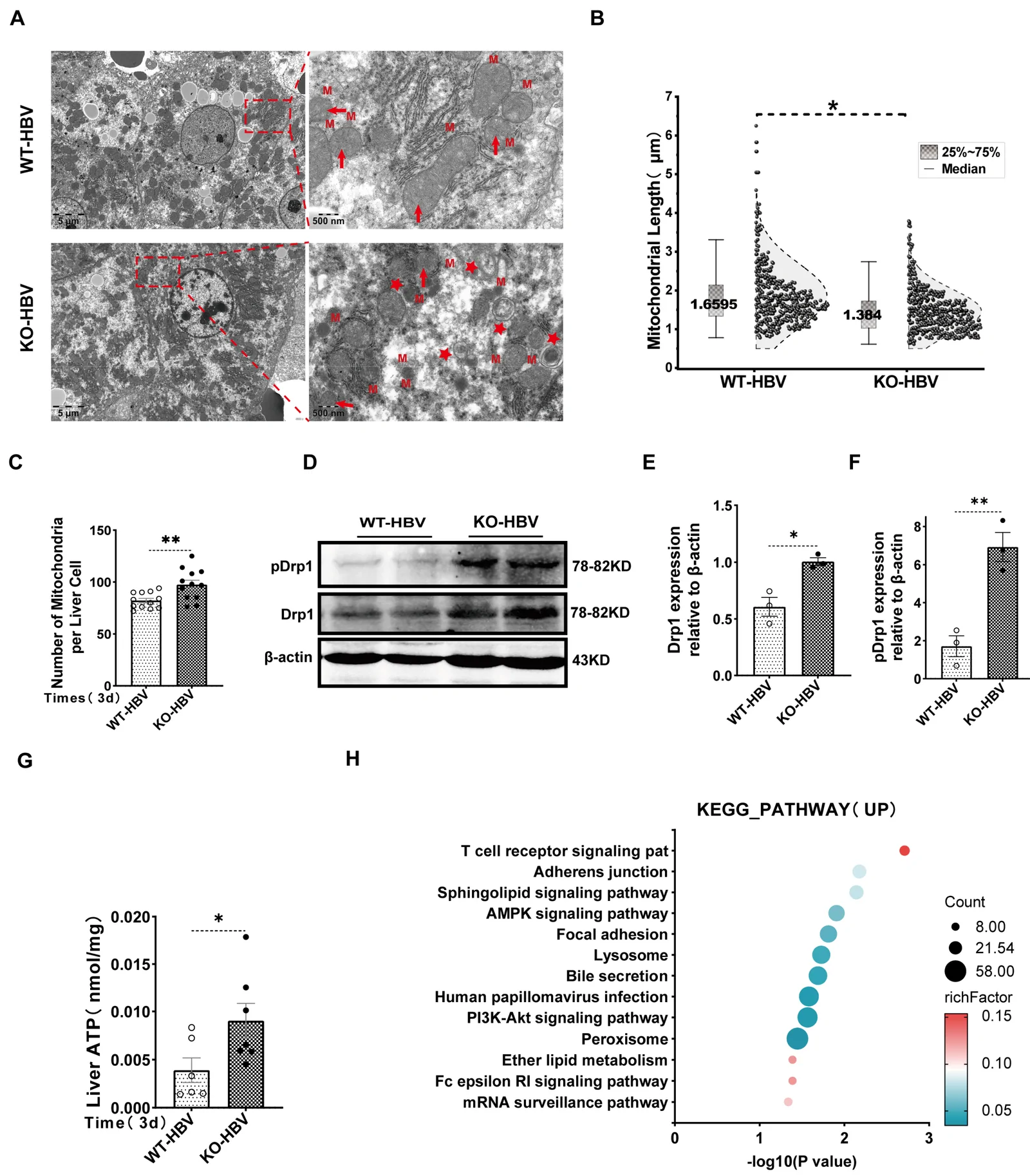
TAGLN knockout disrupts mitochondrial morphology and energy metabolism in HBV-infected mice. (**A**) Representative transmission electron microscopy (TEM) images of mouse liver tissue. M: mitochondria; red arrows: cristae; red stars: autophagic vacuoles. (**B**) Quantification of mitochondrial length per cell derived from panel (**A**). (**C**) Mitochondrial count per hepatocyte from panel (**A**) (Scale bar: 5 μm). (**D**) Western blot analysis of Drp1 and pDrp1 protein expression in mouse liver. (**E**) Quantification of pDrp1 expression levels from panel (**D**). (**F**) Quantification of Drp1 expression levels from panel (**D**). (**G**) Liver ATP levels measured by luminometry. (**H**) KEGG pathway enrichment analysis of upregulated differentially expressed proteins, * *p* < 0.05, ** *p* < 0.01.

Ultrastructural analysis (TEM) of HBV positive mouse livers revealed that, compared to the WT-HBV group, the KO-HBV group exhibited mild mitochondrial edema in hepatocytes, partial dissolution of the outer mitochondrial membrane, reduced or lost cristae, and an increased number of autophagic vacuoles (Figure 5A). Concurrently, the KO-HBV group showed decreased mitochondrial diameter (Figure 5B), an increased mitochondrial number (Figure 5C), and elevated DRP1 protein levels (Figure 5D–F). Liver ATP levels were also higher in the KO-HBV group than in the WT-HBV group (Figure 5G). Collectively, these findings indicate that TAGLN is associated with mitochondrial morphological and functional changes following HBV infection.

To investigate the underlying mechanism, we performed proteomic analysis of mouse liver mitochondrial proteins (Figure S5A). The high inter-sample correlation (Pearson > 0.92, Figure S5B) and tight clustering in PCA (Figure S5C) confirmed data reliability. KEGG pathway enrichment analysis identified 13 significantly upregulated signaling pathways, including the AMPK signaling pathway (Figure 5H). Collectively, these results suggest that although TAGLN deficiency leads to mitochondrial fragmentation, it may activate the AMPK signaling pathway to enhance ATP synthesis, thereby achieving adaptive metabolic reprogramming.

### 2.4. TAGLN Deficiency Correlates with Reduced OPA1 Levels

**Figure 6:**
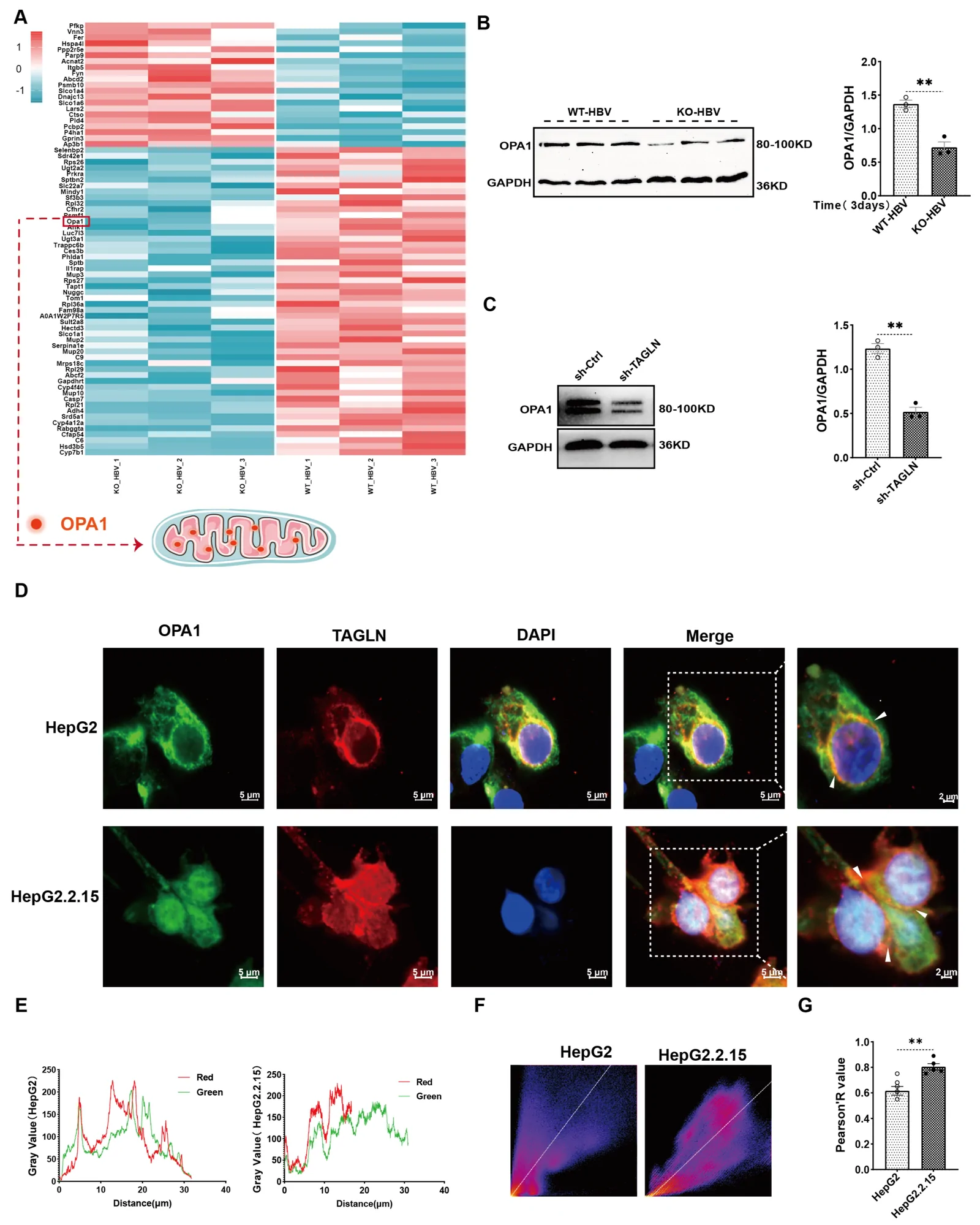
Functional Correlation Between TAGLN and OPA1 in HBV-Infected Cells. (**A**) Hierarchical clustering analysis of differentially expressed mitochondrial proteins. The *y*-axis displays proteins exhibiting significant alterations, with red denoting upregulated proteins, blue representing downregulated proteins, and white indicating proteins with no quantitative changes. Filtering thresholds were set at a fold change >2 and *p* < 0.05. (**B**) Western blot analysis and quantitative assessment of OPA1 protein expression in short hairpin RNA negative control (sh-NC) and sh-TAGLN cells. (**C**) Western blot detection and quantitative analysis of OPA1 protein levels in mouse liver tissues. (**D**) Spatial proximity between OPA1 and TAGLN observed by laser scanning confocal microscopy. OPA1 (green), TAGLN (red), and DAPI (blue) are shown, and the spatial proximity is indicated by arrows. (**E**) Grayscale intensity curves of OPA1 and TAGLN immunofluorescence signals. (**F**) 2D cytofluorograms showing the colocalization of OPA1 (green) and TAGLN (red) in HepG2 and HepG2.2.15 cells. (**G**) Pearson’s correlation coefficient analysis of OPA1-TAGLN colocalization in HepG2 and HepG2.2.15 cells. ** *p* < 0.01.

Mitochondrial proteomic profiling of mouse liver identified OPA1 as a key differentially expressed protein (Figure 6A), which is known to regulate mitochondrial inner membrane dynamics [17]. Western blot analysis confirmed significantly reduced OPA1 expression in the sh-TAGLN group (Figure 6B) and markedly lower liver OPA1 levels in KO-HBV mice compared to the WT-HBV group (Figure 6C), consistent with our omics data. Immunofluorescence further revealed spatial proximity between OPA1 (red) and TAGLN (green) in HepG2 and HepG2.2.15 cells, as indicated by yellow regions in merged images (Figure 6D–F). Notably, the Pearson’s correlation coefficient for these signals was significantly higher in HBV-positive cells than in HBV-negative cells.

### 2.5. OPA1 Expression Correlates with TAGLN Levels and HBV Replication

**Figure 7:**
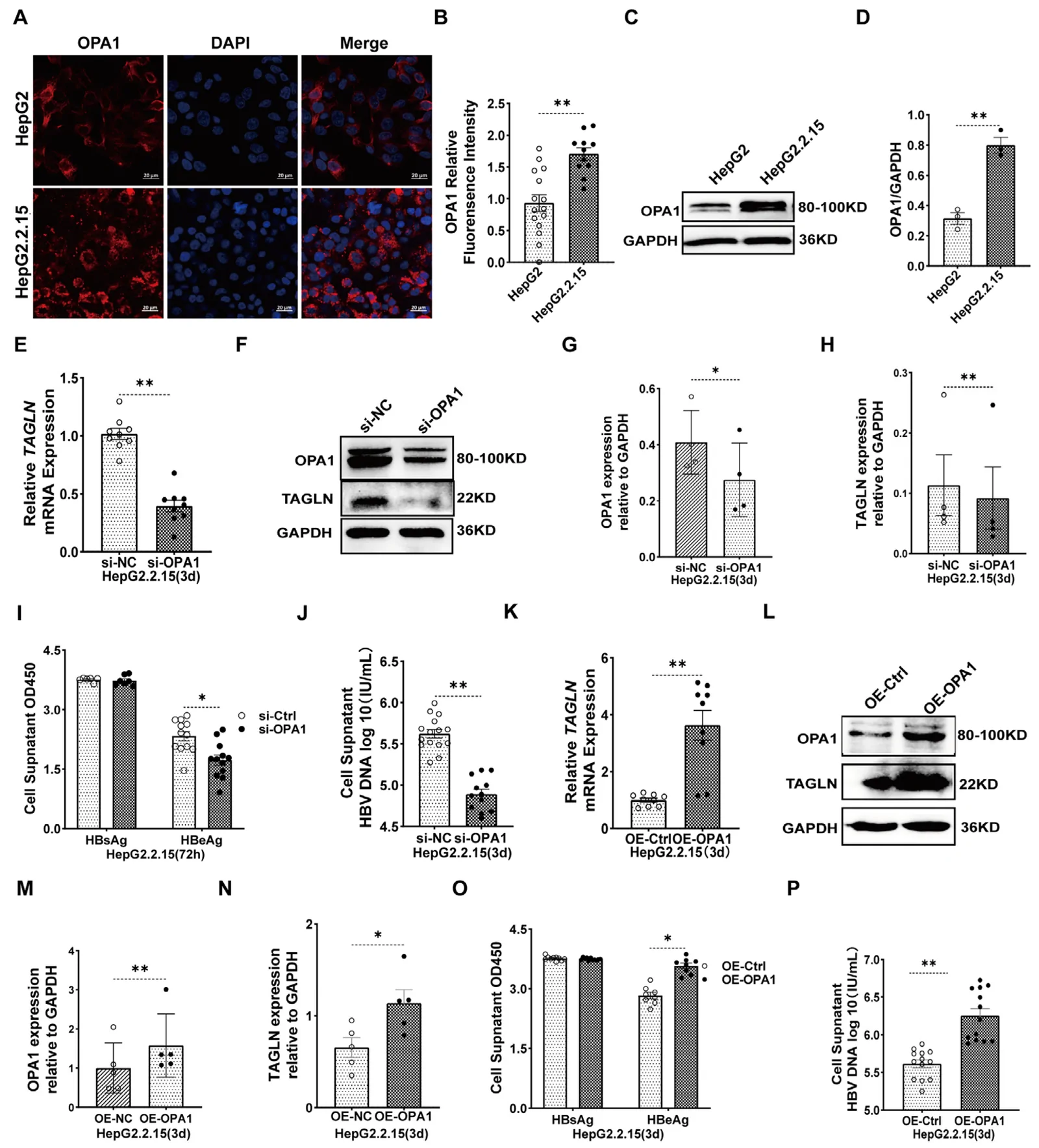
Alterations in OPA1 expression correlate with changes in TAGLN levels and HBV replication. (**A**) Representative immunofluorescence images of OPA1 in HepG2 and HepG2.2.15 cells. OPA1 is shown in red and nuclei were stained with DAPI (blue). (Scale bar: 20 μm). (**B**) Quantitative analysis of OPA1 fluorescence intensity from (**A**). (**C**) Western blot analysis of OPA1 expression. (**D**) Quantification of OPA1 protein levels from (**C**). (**E**) RT-qPCR analysis of TAGLN mRNA levels in si-OPA1-transfected HepG2.2.15 cells. (**F**) Western blot of TAGLN and OPA1 expression in si-OPA1 transfected HepG2.2.15 cells. (**G**) Quantification of OPA1 levels from (**F**). (**H**) Quantification of TAGLN levels from (**F**); (**I**) ELISA of HBsAg and HBeAg secretion in supernatants from si-OPA1 HepG2.2.15 cells. (**J**) Fluorescent probe-based qPCR detection of HBV DNA copies in supernatants of si-OPA1 transfected HepG2.2.15 cells. (**K**) RT-qPCR analysis of TAGLN mRNA levels in OPA1 overexpressing HepG2.2.15 cells. (**L**) Western blot of TAGLN and OPA1 expression in OPA1 overexpressing HepG2.2.15 cells. (**M**) Quantification of OPA1 levels from (**L**). (**N**) Quantification of TAGLN levels from (**L**); (**O**) ELISA of HBsAg and HBeAg secretion in supernatants from OPA1 overexpressing HepG2.2.15 cells. (**P**) Fluorescent probe-based qPCR detection of HBV DNA copy numbers in supernatants of OPA1 overexpressing HepG2.2.15 cells. * *p* < 0.05, ** *p* < 0.01.

This study further examines the relationship between TAGLN and OPA1 during HBV replication. OPA1 expression was markedly elevated in HepG2.2.15 cells compared with HepG2 cells (Figure 7A–D). OPA1 knockdown decreased TAGLN expression at both mRNA and protein levels (Figure 7E–H), and reduced HBeAg secretion and HBV DNA copy numbers (Figure 7I,J). In contrast, OPA1 overexpression increased TAGLN expression (Figure 7K–N) and enhanced HBeAg secretion and HBV DNA levels (Figure 7O,P), without significantly affecting HBsAg. These findings suggest that OPA1 expression is associated with TAGLN levels and HBV replication in HBV-positive cells.

## 3. Discussion

**Figure 8:**
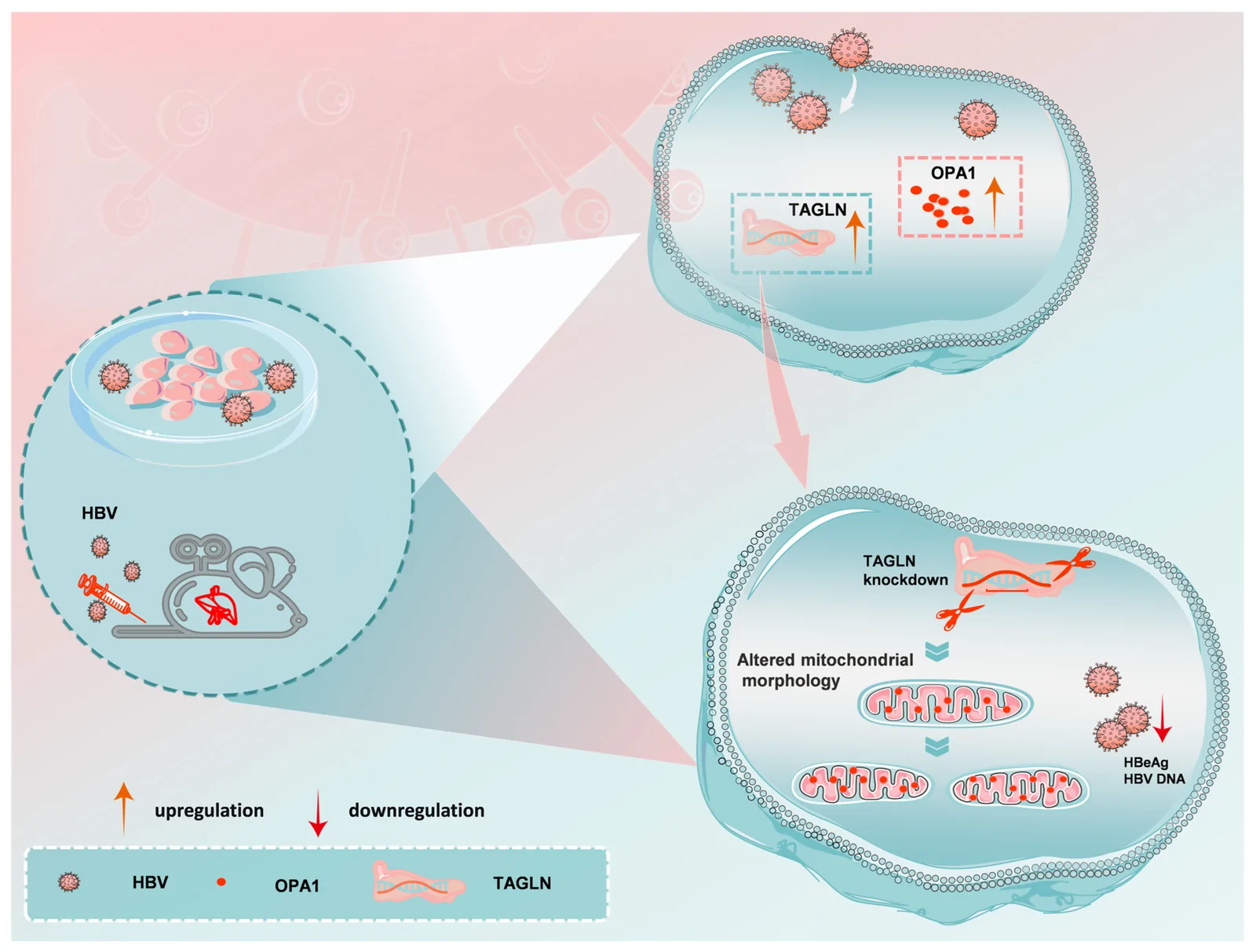
Schematic illustration of TAGLN and OPA1 expression changes associated with HBV replication.

In vitro and in vivo, we observed that HBV infection correlates with increased TAGLN expression, and that TAGLN levels are associated with changes in OPA1 expression and HBV replication (Figure 8), suggesting a link to cytoskeletal remodeling during HBV infection.

For instance, myosin can act as a receptor mediating HBV internalization [16] and interacts with the Hepatitis B virus X (HBx) protein to promote hepatocellular carcinoma (HCC) progression [17]; HBx induces actin reorganization to enhance HCC cell migration [18]; and actin dynamics influence therapeutic drug efficacy in liver cancer [19]. Current research on virus-cytoskeleton interactions is predominantly based on in vitro studies, and the relevance of these findings to in vivo infection models remains unclear. This study demonstrates that HBV infection upregulates TAGLN expression in host cells, showing a positive correlation with HBV DNA load. We speculate that this may relate to the requirement of cytoskeletal involvement during HBV replication [20,21]. As a key actin-binding protein, TAGLN not only participates in cellular regulation [22], but also enhances migratory and invasive capacities in tumor microenvironments [23]. These findings suggest that TAGLN upregulation may favor HBV replication.

The reported role of TAGLN in disease exhibits context-dependency: its depletion typically promotes disease progression [20], while overexpression suppresses inflammation [21]. However, this study found that silencing TAGLN reduces HBV replication levels. This paradox may arise from heterogeneity in model systems, disease stages, and microenvironments. In hepatitis B, TAGLN is recognized as a key biomarker for cirrhosis-related inflammatory immunity [24]. Its deficiency may enhance T-cell responses to promote viral clearance [25], potentially through activation of the T-cell receptor signaling pathway (KEGG analysis) and multiple immune pathways (GO analysis, Figure S5E). These findings suggest that TAGLN functions as a host factor supporting HBV replication, yet its immunoregulatory targets require further mechanistic elucidation.

HBV reactivation, immune dysregulation, and inflammatory responses are intricately intertwined, driving the liver to adapt its metabolic patterns to meet energy demands [26]. This study revealed that silencing TAGLN induces mitochondrial fragmentation. Given the established role of the cytoskeleton in regulating mitochondrial morphology and positioning [27], TAGLN may influence mitochondrial dynamics through the actin network. Intriguingly, although HBV infection typically reduces ATP levels [28], TAGLN deletion elevates ATP levels despite promoting mitochondrial fission. Enrichment of the AMPK signaling pathway suggests a potential metabolic adaptation (Figure 5H), although the underlying mechanisms remain to be elucidated. Overall, TAGLN is associated with altered mitochondrial dynamics and energy metabolism during HBV infection.

Mitochondrial proteomic analysis identified the key differentially expressed protein OPA1. This inner membrane GTPase regulates mitochondrial fusion [29], maintains mitochondrial DNA (mtDNA) stability [30], and suppresses apoptosis [31]. Experimental validation confirmed higher OPA1 expression in the WT-HBV group compared to the KO-HBV group. This difference may be associated with the altered mitochondrial morphology observed upon TAGLN deficiency, which was accompanied by reduced OPA1 expression.

OPA1 has been reported to play diverse roles during viral infection in a virus-specific manner. Its role during viral infection appears virus-specific. Newcastle disease virus inhibits OPA1-mediated fusion to promote replication [32], whereas dengue virus relies on OPA1-associated mitochondrial network integrity to restrict innate immune activation [33]. In the context of HBV, we found that OPA1 expression is higher in HBV-positive cells and that OPA1 levels were positively associated with TAGLN expression and viral replication. Given that OPA1 has been reported to support liver tumor cell metabolism and impair CD8^+^ T-cell surveillance [34,35], the coordinated alterations in TAGLN and OPA1 may be involved in HBV-associated hepatocarcinogenesis, though this requires further investigation.

In summary, our findings indicate that HBV infection upregulates TAGLN expression and is accompanied by alterations in mitochondrial dynamics, energy metabolism, and OPA1 expression. However, some limitations merit consideration. First, although reciprocal changes in expression were observed, we did not determine whether TAGLN and OPA1 interact directly or whether their association occurs at the transcriptional, translational, or post-translational level. Therefore, the upstream–downstream relationship between these proteins remains unresolved, and how they coordinate their expression and activity requires further investigation. Future studies employing high-resolution imaging and biochemical assays will be required to address this question. Second, the cellular and animal models used in this study cannot fully recapitulate the complex hepatic microenvironment in humans. More physiologically relevant liver organoid systems may help clarify the role of OPA1 in TAGLN-associated HBV-related changes. Overall, our results expand the understanding of cytoskeleton–mitochondria crosstalk during HBV infection and suggest that the association between TAGLN and OPA1 may represent a potential target for host-directed antiviral strategies.

## 4. Materials and Methods

### 4.1. Experimental Materials

#### 4.1.1. Plasmids

The pAAV-HBV1.2 plasmid, containing a 1.2-fold HBV genome (genotype D), was generously provided by Prof. Zhenghong Yuan of Fudan University. The pBlue-HBV1.3 plasmid was kindly donated by Prof. Jianguo Wu and Prof. Ke Lan of Wuhan University.

#### 4.1.2. Cell Lines and Culture

The human liver cancer cell lines HepG2, HepG2.2.15 (stably secreting HBV), HepAD38 and THLE-2 were preserved in our laboratory. Among these, HepG2 and THLE-2 do not secrete viruses. The sh-TAGLN cell line (TAGLN-knockdown HepG2.2.15 cells) was constructed in our laboratory [12]. All cells were cultured in DMEM/F12 (1:1) complete medium supplemented with 10% fetal bovine serum and 1% penicillin (100 IU/mL) and maintained in a 5% CO_2_ incubator at 37 °C with saturated humidity. HepG2.2.15 cells were cultured with 400 μg/mL G418 to maintain viral titer, while HepAD38 cells were cultured with 1 μg/mL tetracycline.

#### 4.1.3. Animal Models and Ethics

C57BL/6 mice (4–6 weeks old, male, body weight 15 ± 2 g) were purchased from the Laboratory Animal Center of Zunyi Medical University (License No. SYXK (Qian) 2021-0004, Zunyi, China). TAGLN-knockout (TAGLN-KO) mice (C57BL/6J background, 4–6 weeks old, male, body weight 15 ± 2 g) were obtained from Saiye (SCXK (Su) 2022-0016). All mice were housed in a specific pathogen-free environment with controlled temperature, humidity, and a 12 h light/dark cycle. Mice were acclimatized for 7 days prior to experiments. All procedures were conducted in compliance with the Guide for the Care and Use of Laboratory Animals and approved by the Ethical Review Committee for Animal Experiments of Zunyi Medical University (Approval No. ZMU21-2303-189).

### 4.2. Modeling of HBV Infection

THLE-2 cells: Seeded in 24-well plates (diameter: 16 mm) at 1.0 × 10^5^ cells/well. After 24 h, the cells were transfected with varying doses of pBlue-HBV1.3. Supernatants and cellular proteins were collected 72 h post-transfection.

HepAD38 cells: Treated with tetracycline for 7 days prior to withdrawal. Cells were then seeded in 24-well plates (diameter: 16 mm) at 1.0 × 10^5^ cells/well. Supernatants and proteins were harvested at 1, 3, and 7 days post-withdrawal.

Hydrodynamic injection (HDI) mouse model:

An HBV-infected C57BL/6J mouse model was established using the HDI method [36]. Animals were randomly assigned to groups using a computer-generated random sequence. Briefly, a phosphate-buffered saline (PBS) solution containing 20 μg of the pAAV-HBV1.2 plasmid was rapidly injected into the tail vein of mice within 10 s. The injection volume was calculated as 10% of the mouse’s body weight (approximately 15 g). The control group received an equivalent volume of PBS containing an empty pAAV vector. After injection, mice were warmed and monitored daily for general condition, body weight, and injection site reactions until sample collection. Samples were collected at 3, 8, and 13 days post-injection. Mice were anesthetized by intraperitoneal injection of sodium pentobarbital (40 mg/kg body weight, prepared as a 1% solution in sterile saline) and after anesthesia, blood (0.5 mL) was collected from the retroorbital venous plexus for serum separation. Subsequently, animals were humanely euthanized by cervical dislocation. Liver tissues were then harvested immediately. A portion of the liver tissues was fixed in 10% neutral formalin, another portion was fixed in glutaraldehyde, and the remaining liver tissues were stored at −80 °C for further analysis.

### 4.3. ELISA for the Detection of Hepatitis B Surface Antigen (HBsAg) and Hepatitis B e Antigen (HBeAg)

The concentrations of HBeAg and HBsAg in cell supernatants and mouse serum were quantified using a commercial ELISA kit (Zhongshan Biologicals, S10910123, Zhongshan, China) following the manufacturer’s protocol. Absorbance measurements were performed at 450 nm using a microplate reader (BioTek, Winooski, VT, USA) to determine the optical density (OD) values for each well.

### 4.4. Quantification of HBV DNA by PCR-Fluorescent Probe Method

HBV DNA was isolated from cell culture supernatants and murine serum utilizing the Hepatitis B Virus Nucleic Acid Assay Kit (DAAN GENE, DA0030, Guangzhou, China) following the manufacturer’s protocol. Subsequently, the extracted DNA was amplified and quantitatively analyzed through real-time PCR with fluorescence-based probe detection.

### 4.5. Hematoxylin and Eosin (HE) Staining of Mouse Liver Tissues

Liver tissues were fixed in 10% neutral buffered formalin, dehydrated, paraffin-embedded, and sectioned (5 μm). Sections were baked at 60 °C for 2 h, deparaffinized in xylene (2 × 10 min), and rehydrated through graded ethanol. After staining with hematoxylin (differentiated in 1% acid alcohol and blued in ammonia ethanol) and eosin (10 min), slides were dehydrated in ethanol, cleared in xylene, and mounted with neutral resin. Histopathological changes were examined under a light microscope (Leica Co., Ltd., Wetzlar, Germany).

### 4.6. RT-qPCR Method to Determine the Transcript Levels of Related Genes

Mouse livers were collected at various time points, and total RNA was extracted using Trizol reagent (TaKaRa, 9108, Tokyo, Janpan). cDNA was synthesized using PrimeScript RT Master Mix (TaKaRa, RR036A) according to the manufacturer’s protocol, followed by quantitative RT-PCR using gene-specific primers (all synthesized by Sangon Biotech, Shanghai, China): TAGLN (Forward: 5′-CTTTGGGCAGTTTGGCTGTG-3′, Reverse: 5′-GCTCCTGGGCTTTCTTCATAAAC-3′), HBV DNA (Forward: 5′-GGAGTGTGGATTCGCACTCCTC-3′, Reverse: 5′-TTCTTCTTCTAGGGGACCTGCCT-3′), and Glyceraldehyde 3-phosphate dehydrogenase (GAPDH, Forward: 5′-GGTTGTCTCCTGCGACTTCA-3′, Reverse: 5′-TGGTCCAGGGTTTCTTACTCC-3′) as the internal control. Amplification was performed on a CFX96 Real-Time PCR System (Bio-Rad, Tokyo, Janpan), with relative gene expression analyzed using the 2^−ΔΔCt^ method normalized to GAPDH.

### 4.7. Western Blot Analysis of Target Protein Expression

Cells and mouse liver samples were collected and lysed using RIPA buffer supplemented with protease inhibitors (Solarbio, Beijing, China). After 30 min of lysis, the samples were centrifuged at 12,000× *g* for 10 min at 4 °C. The supernatant was collected, and protein concentrations were determined using the bicinchoninic acid (BCA) assay.

Proteins were separated by SDS-PAGE and transferred onto polyvinylidene difluoride (PVDF) membranes. The membranes were blocked with 5% BSA (Solarbio, SW3015, Beijing, China) at room temperature for 2 h. Subsequently, the membranes were incubated with primary antibodies against TAGLN (BOSTER, A03962-2, Wuhan, China), OPA1 (Proteintech, 66583-1, Tokyo, Janpan), and GAPDH (Beyotime, AF1186, Shanghai, China) overnight at 4 °C. After washing with TBST, the membranes were incubated with a sheep anti-rabbit secondary antibody (Beyotime, A0208) at room temperature for 2 h. Specific protein bands were visualized using ECL reagent (Solarbio, PE0010) following TBST washes.

### 4.8. Immunohistochemical Detection of HBsAg and TAGLN Expression

Mouse livers were fixed in 10% neutral formalin, dehydrated, embedded in paraffin, and sectioned at a thickness of 5 μm. The sections were deparaffinized in xylene and rehydrated. Antigen retrieval was performed using high-pressure treatment with 10 mM citrate buffer (pH 6.0). Endogenous peroxidase activity was quenched by incubating the sections in 3% H_2_O_2_. After blocking with 5% sheep serum for 1 h, the sections were incubated overnight at 4 °C with primary antibodies, including rabbit anti-goat (Bioss, bs-1557G, Beijing, China) and TAGLN (BOSTER, A03962-2). Subsequently, the sections were incubated with secondary antibodies, rabbit anti-sheep (Solarbio, SE238) and sheep anti-rabbit (Beyotime, A0208), for 1 h. Color development was achieved using a DAB kit (zsgb-bio, ZLI-9018, Beijing, China), and the sections were counterstained with hematoxylin. Finally, the sections were dehydrated, mounted, and observed under a microscope to assess the expression of HBsAg and TAGLN in mouse liver tissues.

### 4.9. TEM (Transmission Electron Microscopy) Analysis of Mitochondrial Morphology

Mouse tissues fixed in 2.5% glutaraldehyde were rinsed with PBS, followed by post-fixation in 1% osmium tetroxide for 1 h. Dehydration was performed by sequentially immersing the tissues in ethanol solutions of increasing concentrations (70%, 95%, and 100%) at room temperature. The tissues were then treated with propylene oxide for 20 min at 4 °C. Subsequently, the tissues were embedded in epoxy resin and sectioned using a Leica EM UC6 ultramicrotome (Leica Microsystems, Wetzlar, Germany). Mitochondrial morphology was observed using transmission electron microscopy (Hitachi, Tokyo, Japan).

### 4.10. Luminometer Determination of Adenosine Triphosphate (ATP) Levels

HepG2 and HepG2.2.15 cells were seeded into 24-well plates at a density of 1.0 × 10^5^ cells per well. After 72 h of culture, the medium was aspirated. Alternatively, equal weights of mouse liver tissues were weighed to measure ATP levels using an ATP assay kit (Beyotime, S0026). The absorbance of the samples was quantified using a Luminometer chemiluminescence reader (Berthold Technologies, Bad Wildbad, Germany), and ATP concentrations were calculated based on a standard curve. Protein concentrations in the samples were determined using the BCA method, and ATP levels were normalized to protein content, expressed as nmol/mg protein.

### 4.11. Mitochondrial Proteomics Analysis

Mitochondrial proteins were extracted from mouse liver tissues using a Mitochondrial Extraction Kit (Solarbio, SM0020). The enrichment efficiency was confirmed by Western blot analysis for the mitochondrial marker TOMM20. Protein concentration was quantified by SDS-PAGE. For proteomic analysis, proteins were lysed in SDS-DTT-Tris buffer and digested with trypsin using the filter-aided proteome preparation method, followed by desalting. The resulting peptides were reconstituted in 0.1% formic acid and analyzed by liquid chromatography-tandem mass spectrometry using 4D-Labelfree technology. Acquired spectra were processed with MaxQuant software (version 1.6.14) for protein identification and label-free quantification. The mass spectrometry proteomics data have been deposited to the ProteomeXchange Consortium via the iProX partner repository with the dataset identifier PXD063056.

### 4.12. Bioinformatic Analysis

#### 4.12.1. Differentially Expressed Proteins Screening (DEPs)

DEPs were screened using fold-change >2 and *p* value < 0.05 (*t*-test). Hierarchical clustering analysis was performed, and heatmaps were generated using Cluster 3.0 (Version 1.59) and Java Treeview (Version 1.2.0). The Euclidean distance was selected as the similarity metric, and the average linkage clustering algorithm, based on the centroids of observations, was employed. Data underwent Z score standardization to eliminate differences in scale. The heatmap displays protein expression patterns using a color gradient. Ultimately, the mitochondrial fusion protein OPA1 was identified as a key differentially expressed protein.

#### 4.12.2. Kyoto Encyclopedia of Genes and Genomes (KEGG) Annotation

Differentially expressed proteins were annotated using KOBAS 3.0 software against the KEGG database. Enrichment analysis was performed via Fisher’s exact test with Benjamini–Hochberg correction, and pathways with an adjusted *p* < 0.05 were considered significantly enriched. Visualization of the enrichment results (bubble plots) was conducted using R software (v4.3.3; R Foundation for Statistical Computing, Vienna, Austria).

#### 4.12.3. Principal Component Analysis (PCA)

Data were first normalized to total peak intensity. Subsequently, the normalized data were imported into SIMCA-P (v14.1, Umetrics, Umeå, Sweden) for Pareto scaling and PCA to evaluate inter- and intragroup variability. Analysis results were visualized using Origin 2021 (OriginLab Corporation, Northampton, MA, USA).

### 4.13. Cell Transfection with OPA1 RNA (si-OPA1) and Overexpression OPA1 Plasmid (OE-OPA1)

HepG2.2.15 cells were seeded into 24-well plates (approximately 16 mm in diameter) at a density of 1.0 × 10^5^ cells per well. After 24 h, the medium was replaced with antibiotic-free medium. and cells were transfected with Lipo6000™ Transfection Reagent (Beyotime, C0526, Shanghai, China) following the manufacturer’s protocol. Transfected constructs included: si-OPA1 (sense: 5′-GGACCUUAGUGAAUAUAAA-3′, antisense: 5′-UUUAUAUUCACUAAGGUCC-3′) and negative control siRNA (si-NC; sense: 5′-UUCUCCGAACGUGUCACGUTT-3′, antisense: 5′-ACGUGACACGUUCGGAGAATT-3′) (both from Sangon Biotech); and OE-OPA1 (pML-EGFP-OPA1) plus its empty vector control (OE-CTRL; pML-EGFP) (both from Zomanbio, ZK29840, Beijing, China). Supernatants and cells were collected 72 h post-transfection.

### 4.14. Laser Scanning Confocal Microscopy to Observe Cellular Mitochondrial

After discarding the medium, sh-NC (HepG2.2.15) and sh-TAGLN (HepG2.2.15) cells were washed with PBS. The cells were then incubated with Mito-Tracker (100 nM, Invitrogen, M22426, Carlsbad, CA, USA), pre-warmed to 37 °C, for 30 min at 37 °C in the dark. Following incubation, the cells were washed with pre-warmed serum-free medium. Mitochondrial morphology was visualized using a laser scanning confocal microscope (Carl Zeiss Microscopy GmbH, Oberkochen, Germany).

### 4.15. Cell Proliferation Assay by CCK-8 (Cell Counting Kit-8)

sh-NC and sh-TAGLN cells were seeded in 96-well plates at a density of 1 × 10^4^ cells per well and cultured for 48 h. Subsequently, 10 μL of CCK-8 solution was added to each well, followed by incubation at 37 °C for 2 h. Absorbance was measured at 450 nm using a microplate reader (Multiskan GO; Thermo Fisher Scientific Oy, Vantaa, Finland). Cell viability (%) = (OD experimental − OD blank)/(OD control − OD blank) ×100%.

### 4.16. Immunofluorescence Staining

#### 4.16.1. Immunofluorescence Spatial Proximity Analysis of OPA1 and TAGLN

HepG2 and HepG2.2.15 cells were fixed with 4% paraformaldehyde at room temperature for 30 min, permeabilized with 0.25% Triton X-100 for 15 min, and then blocked with 1% BSA for 1 h. Subsequently, the cells were incubated with a mixture of primary antibodies (OPA1: MCE HY-P80255; TAGLN: Santa Cruz sc-53932) overnight at 4 °C. After washing with PBS, the cells were incubated with a mixture of fluorescent secondary antibodies (AF594-conjugated goat anti-mouse: Bioss bs-0296G; Alexa Fluor 488-conjugated goat anti-rabbit: abcam ab150077) in the dark for 1 h. After being washed with PBS and stained with DAPI, the slices were sealed with an anti-fluorescence quenching sealer and then the results were observed under a laser scanning confocal microscope.

#### 4.16.2. Immunofluorescence Staining for OPA1

HepG2 and HepG2.2.15 cells were fixed with 4% paraformaldehyde for 30 min at room temperature, permeabilized with 0.25% Triton X-100 for 15 min, and blocked with 1% BSA for 1 h. The cells were then incubated overnight at 4 °C with the primary antibody OPA1 (Proteintech, 66583-1-Ig). After washing with PBS, the cells were incubated with Alexa Fluor 594-conjugated sheep anti-mouse secondary antibody (Bioss, bs-0296G-AF594) for 1 h at room temperature. Nuclei were counterstained with DAPI, and the results were visualized using a laser scanning confocal microscope.

### 4.17. Data Analysis

Statistical analysis was performed using GraphPad Prism 9.0 (GraphPad Software, San Diego, CA, USA). Data are presented as mean ± SEM. Normality was assessed prior to parametric testing. Comparisons between two independent groups were conducted using an unpaired two-tailed Student’s *t*-test. Comparisons among multiple groups were analyzed by one-way ANOVA followed by Tukey’s post hoc test. For correlation analyses of non-normally distributed variables (Figure 1 and Figure 2), Spearman’s rank correlation was used. In Figure 6, Pearson’s correlation coefficient (R) was calculated for each confocal image using ImageJ 1.54p (National Institutes of Health, Bethesda, MD, USA), and R values between two independent groups were compared using an unpaired two-tailed Student’s *t*-test. A *p* value < 0.05 was considered statistically significant.

## 5. Conclusions

This study indicates that TAGLN is associated with changes in mitochondrial dynamics, cellular energy metabolism, and HBV replication. An association between TAGLN and OPA1 expression was also observed and correlated with HBV replication levels. These findings expand the current understanding of HBV–host interactions at the mitochondrial level and suggest that TAGLN may be linked to mitochondrial alterations during HBV replication.

## Data Availability

Supplementary Figures S1–S5 and their legends are provided in the Supplementary Materials. The datasets supporting the conclusions of this study are available from the corresponding author upon reasonable request. The mitochondrial proteomics data have been deposited in the ProteomeXchange Consortium repository (dataset identifier: PXD063056).

## Supplementary Materials

The following supporting information can be downloaded at: https://www.mdpi.com/article/10.3390/ijms27167338/s1.

## Abbreviations

AMPK	AMP-activated protein kinase
ATP	adenosine triphosphate
CCK-8	Cell Counting Kit-8
Ctrl	control
DEPs	differentially expressed proteins
Drp1	dynamin-related protein 1
ELISA	enzyme-linked immunosorbent assay
FASP	filter-aided proteome preparation
GAPDH	glyceraldehyde 3-phosphate dehydrogenase
GO	Gene Ontology
HBeAg	Hepatitis B e antigen
HBsAg	Hepatitis B surface antigen
HBV	Hepatitis B virus
HBx	Hepatitis B virus X protein
HCC	hepatocellular carcinoma
HDI	hydrodynamic injection
HE	hematoxylin and eosin
IHC	immunohistochemistry
KEGG	Kyoto Encyclopedia of Genes and Genomes
KO	knockout
LC-MS/MS	liquid chromatography-tandem mass spectrometry
mtDNA	mitochondrial DNA
OPA1	optic atrophy 1
PCA	principal component analysis
PCR	polymerase chain reaction
RT-qPCR	reverse transcription quantitative real-time PCR
sh-NC	short hairpin RNA negative control
si-NC	small interfering RNA negative control
OE-CTRL	overexpression control plasmid
OE-OPA	overexpression OPA1 plasmid
si-OPA1	small interfering RNA targeting OPA1
sh-TAGLN	short hairpin RNA targeting TAGLN
TAGLN	Transgelin
TEM	transmission electron microscopy
WT	wild-type
