# Supplementary material for: Altered TAGLN Expression During HBV Replication Correlates with OPA1 Levels
Source: Int J Mol Sci. 2026 Aug 17;27(16):7338. doi: 10.3390/ijms27167338 (PMC13513760; doi:10.3390/ijms27167338)
Supplement: Supplementary file 1 [file ijms-27-07338-s001.zip › ijms-4360314-supplementary.pdf]

Supplementary Figures

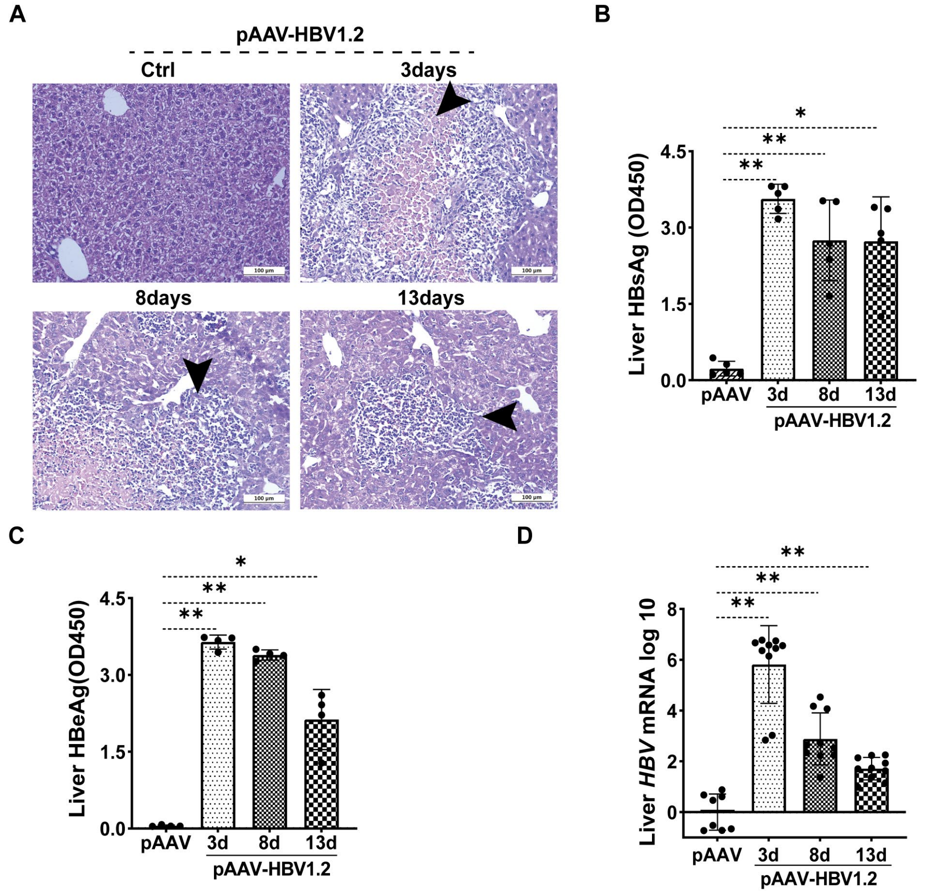

Figure S1 Liver histopathology and HBV marker expression in the HDI mouse model. A: HE staining of mouse liver tissue (Scale bar: 100 μm); B: ELISA detection of HBsAg secretion levels in mouse liver tissues. C: ELISA detection of HBeAg secretion levels in mouse liver tissues. D: RT-qPCR quantification of HBV mRNA expression levels in mouse livers, *$p < 0.05$, **$p < 0.01$.

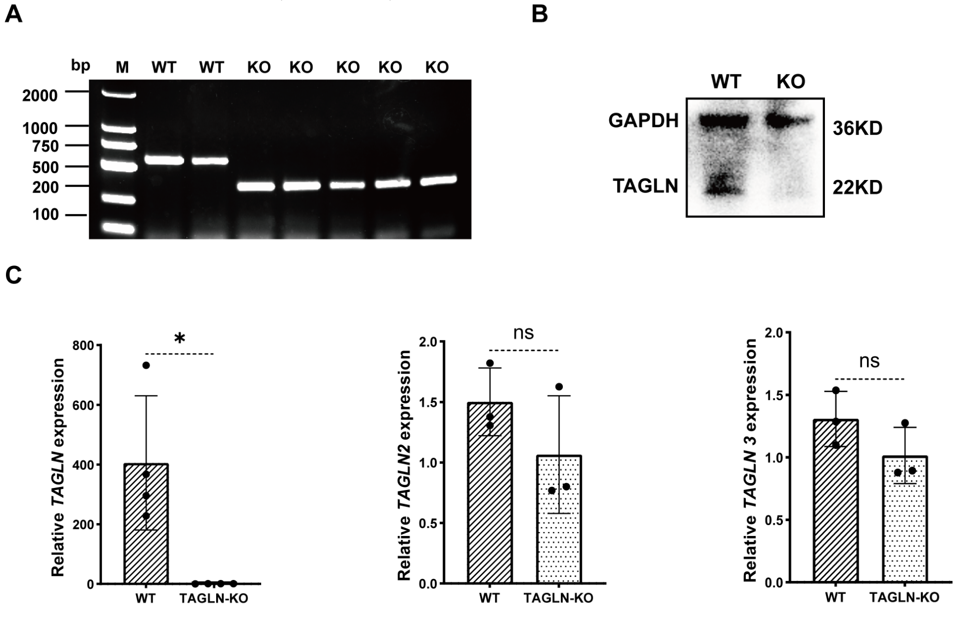

Figure S2 Validation of TAGLN knockout efficiency in mice. A: Gel electrophoresis image of genotyping for TAGLN-KO mice. Lane M: DNA marker; the first two lanes: WT mouse samples; the remaining lanes: KO

mouse samples. B: Western Blot detection of TAGLN expression in the liver of TAGLN-KO mice, C: RT-qPCR analysis of TAGLN mRNA expression levels in mouse liver tissues. *$p < 0.05$, **$p< 0.01$.

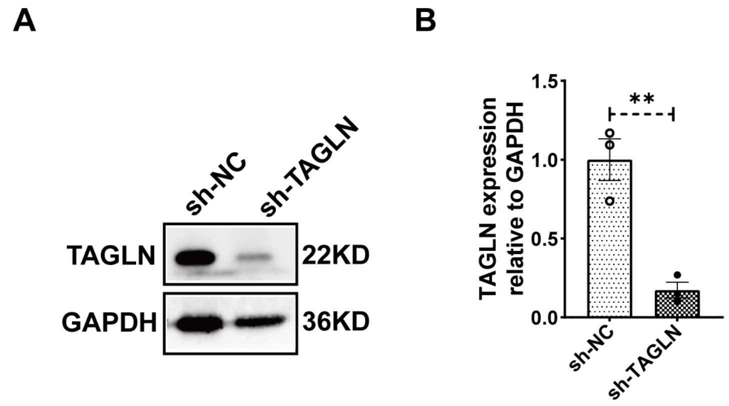

Figure S3 Validation of TAGLN knockdown in HepG2.2.15 cells. A: Western Blot analysis showing the protein expression levels of TAGLN following its knockdown in HepG2.2.15 cells; Quantification of the TAGLN levels from A, **$p< 0.01$.

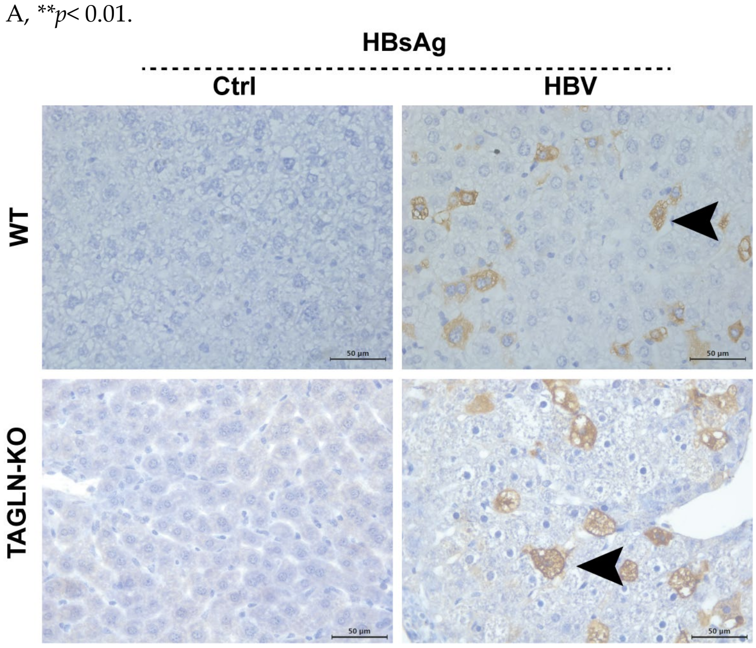

Figure S4 HBsAg expression in the liver of HDI mice. Immunohistochemical analysis of HBsAg expression in mouse livers (Arrows indicate positive signals; Scale bar: 50 μm)

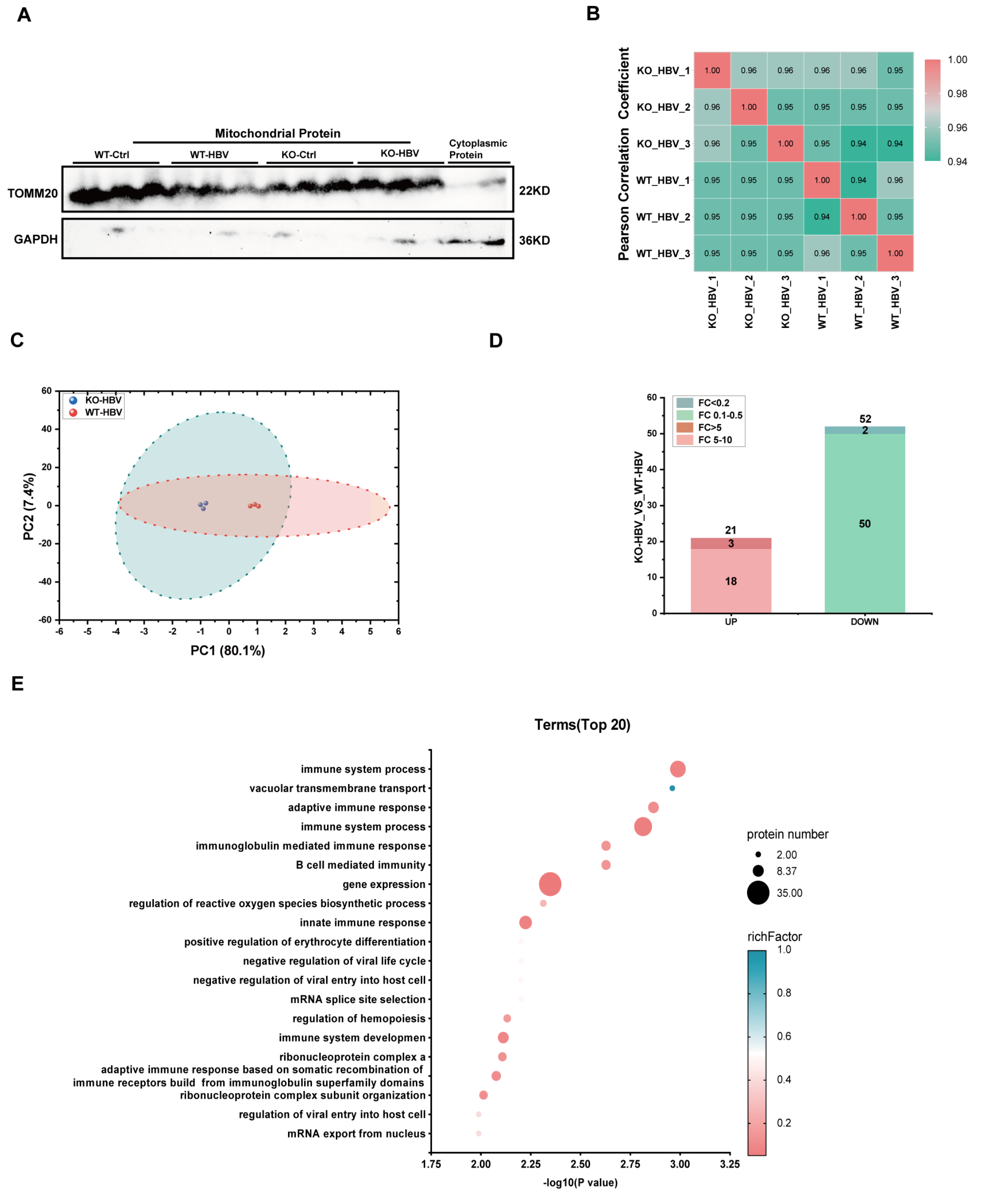

Figure S5 Differential analysis of mitochondrial protein expression in mouse liver. A: Mitochondrial protein purity assessment. TOMM20 was highly enriched in mitochondrial fractions with minimal GAPDH contamination, while cytoplasmic fractions showed low TOMM20 but high GAPDH expression. B: Pearson correlation analysis of intergroup relationships (1: strong positive correlation; -1: strong negative correlation; 0: no correlation). C: PCA analysis demonstrating distribution patterns of samples from different experimental groups. Each point represents an individual sample, with distinct colors indicating different treatment groups. D: Screening of differentially expressed proteins (highlighted in dark color for FC>2 and P<0.05). E: GO enrichment analysis of differential proteins (x-axis: -log10 p Value, *p*<0.05).
